# Waxy pink facial lesion on an elderly man

**DOI:** 10.1016/j.jdcr.2024.11.020

**Published:** 2024-11-29

**Authors:** Gabrielle Minta, Sonal Muzumdar, Campbell L. Stewart, Ali Banki

**Affiliations:** a Frank H Netter MD School of Medicine, North Haven, Connecticut; b Department of Dermatology, University of Connecticut Health Center, Farmington, Connecticut; c Banki Dermatology and Cosmetic Center, Glastonbury, Connecticut

**Keywords:** amyloidosis, nodular amyloid

## Case

**Fig 1:**
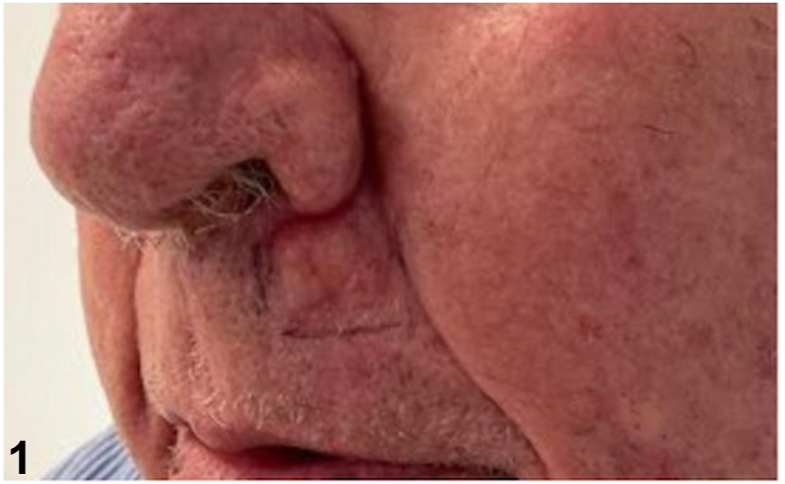

**Fig 2:**
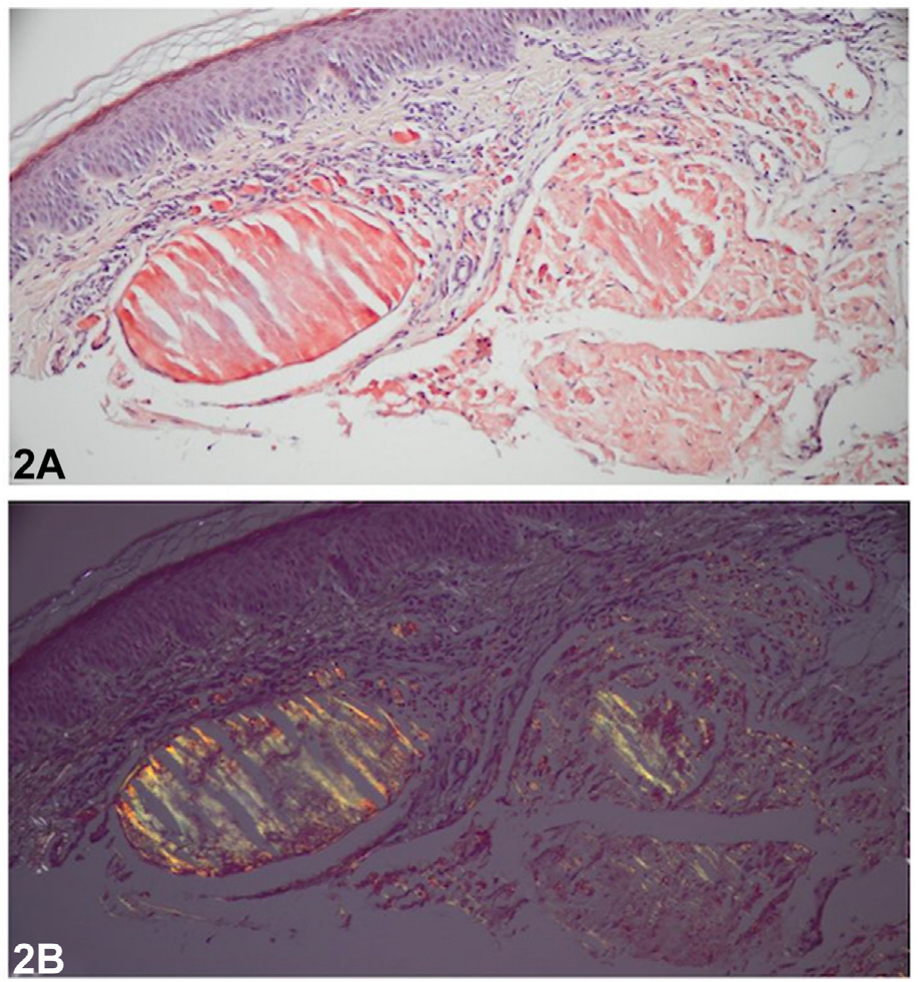

**Fig 3:**
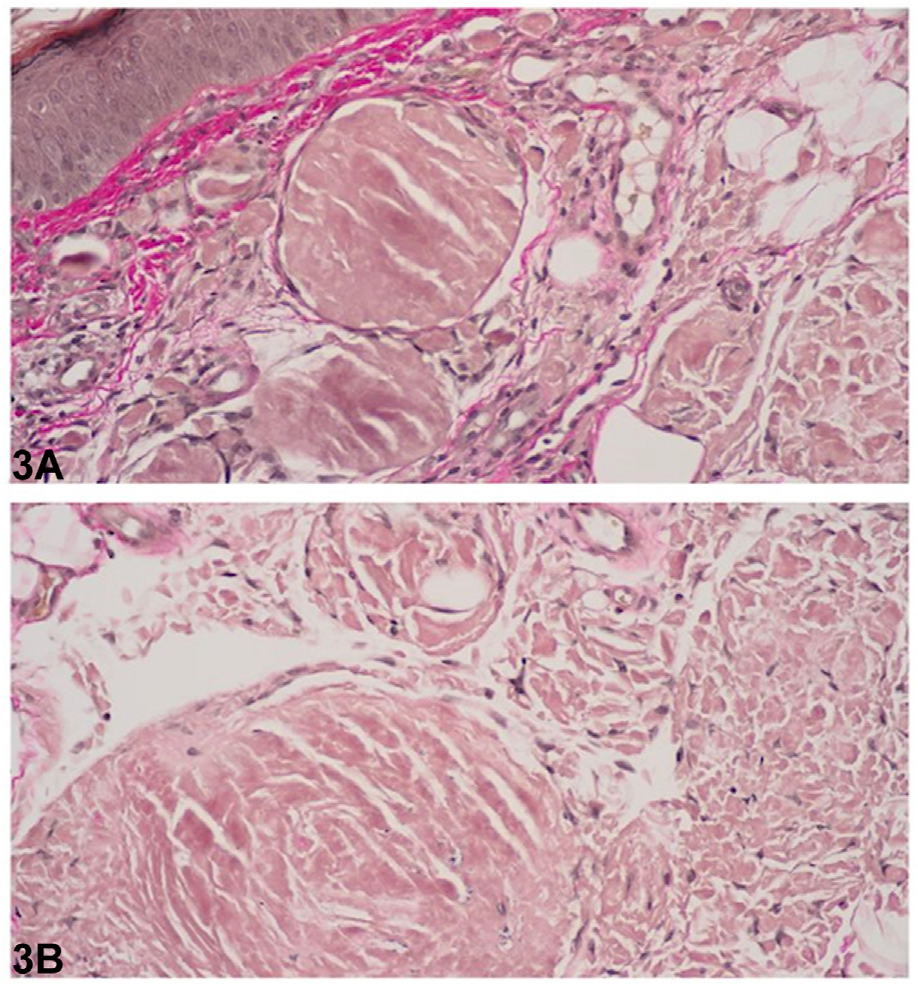

An 86-year-old male presented to the clinic with a pink, waxy plaque of the left upper cutaneous lip (Fig 1). Review of systems was negative and his medical, surgical, and family history were noncontributory. Histology demonstrated dermal deposition of ovoid aggregates composed of fragmented homogenous eosinophilic material. Congo red stain was positive and demonstrated diffuse apple green birefringence on polarization (Fig 2). Van Gieson stain highlighted the deposits in red (Fig 3). Periodic acid-Schiff-diastase stain was negative. Plasma cells were sparse; however, there was evidence of light chain restriction with an inverted lambda to kappa ratio of approximately 2:1 (not shown).

**Question 1: What is the most likely diagnosis?**

A. Colloid milium
B. Hyaluronic acid filler reaction
C. Lipoid proteinosis (LP)
D. Nodular amyloidosis
E. Sarcoidosis

**Answers:**

A. Colloid milium – Incorrect. Colloid milium mimics nodular amyloidosis but differs in Van Gieson staining (yellow) and clinical presentation with small, dome-shaped papules on sun-exposed areas.^1^ Both can be positive for Congo red and colloid milium can show mild focal apple green birefringence on polarization. Colloid milium electron microscopy shows short, branching, wavy filaments and remains the gold standard for differentiation.
B. Hyaluronic acid filler reaction – Incorrect. Reactions to hyaluronic acid filler commonly affect the lips and perioral area, presenting as cystic granulomas, occurring weeks to years after treatment. Histology shows basophilic material surrounded by multinucleated giant cells.
C. Lipoid proteinosis (LP) – Incorrect. LP results in the deposition of hyaline material in the skin, brain, and organs.^2^ LP presents with a hoarse cry in infancy, vesicles, and pitted scars. Later lesions include characteristic beaded papules on the eyelids (moniliform blepharosis) and verrucous papules and plaques on extensor extremities.^2^ In contrast to nodular amyloidosis, LP is periodic acid-Schiff positive, diastase resistant, and Congo red is negative or weakly positive.
D. Nodular amyloidosis – Correct. Nodular amyloidosis involves light chain immunoglobulin light chain deposition, seen as pink, waxy nodules clinically.^3^ Nodular amyloidosis stains positive for Congo red, thioflavin T, and methyl violet and appears as nonbranching filaments under an electron microscope. Polarization of Congo red staining shows apple-green birefringence. Van Gieson staining of nodular amyloid is red in contrast to the yellow seen in colloid milium. Plasma cells are normally present and demonstrate light chain restriction, in contrast to systemic amyloidosis.
E. Sarcoidosis – Incorrect. Sarcoidosis is a multisystem disease with a wide range of dermatologic manifestations, including subcutaneous nodules which tend to be firm, mobile, and found on the extremities. Histopathology shows non-caseating granulomas.

**Question 2: What is the recommended workup for this patient?**

A. Bacterial and fungal tissue culture
B. Chest X-ray and serum calcium
C. Head computed tomography/magnetic resonance imaging
D. Serum protein electrophoresis (SPEP), urine protein electrophoresis (UPEP), immunofixation electrophoresis (IFE), antinuclear antibodies (ANAs)
E. Serum uric acid, erythrocyte sedimentation rate

**Answers:**

A. Bacterial and fungal tissue culture – Incorrect. This test would be negative in nodular amyloidosis.
B. Chest X-ray and serum calcium – Incorrect. This test would be an appropriate workup for sarcoidosis.
C. Head computed tomography/magnetic resonance imaging – Incorrect. This test would be part of the appropriate workup for lipoid proteinosis.
D. Serum protein electrophoresis (SPEP), urine protein electrophoresis (UPEP), immunofixation electrophoresis (IFE), antinuclear antibodies (ANAs) – Correct. Nodular amyloidosis has a variable risk of progression to systemic amyloidosis, although true rates are likely lower than previously described. This risk is likely decreased if there is no evidence of clinical or laboratory findings at the time of diagnosis.^4^^,^^5^ SPEP, UPEP, IFE, and ANAs can screen for multiple myeloma and autoimmune connective tissue disease, respectively, both of which are associated with systemic amyloidosis. Nodular amyloidosis has been found to be associated with Sjögren’s syndrome, and additional testing for anti-Ro and anti-La may be indicated based on patient demographics, associated symptoms, and location of lesions.
E. Serum uric acid, erythrocyte sedimentation rate – Incorrect. This would be the appropriate workup for gout.

**Question 3: What is the appropriate treatment for this patient?**

A. Cryotherapy
B. Oral cyclosporine
C. Systemic retinoids
D. Surgical intervention
E. UVA or UVB radiation therapy

**Answers:**

A. Cryotherapy – Incorrect. Cryotherapy has not been shown to be effective in the treatment of nodular amyloidosis. One case report demonstrated negative outcomes, with minor hemorrhage resulting from cryotherapy treatment for nodular amyloidosis.^3^
B. Oral cyclosporine – Incorrect. Cyclosporine has been shown to decrease the amount of proinflammatory cytokines leading to improvement in appearance and pruritis of lichen amyloidosis^3^ but has not been shown to be effective in the treatment of nodular amyloidosis.
C. Systemic retinoids – Incorrect. While oral acitretin and etretinate have been shown to improve lesion appearance and pruritis in lichen amyloidosis,^3^ they have not been shown to be effective for nodular amyloidosis.
D. Surgical intervention – Correct. Surgical intervention is the preferred treatment for nodular amyloidosis. Curettage, surgical excision, and dermabrasion have all shown to be effective for nodular amyloidosis with complete clearance.^3^
E. UVA or UVB radiation therapy – Incorrect. UVA or UVB radiation treatment, in combination with topical therapies, has variable improvement on appearance and pruritis in both lichen and macular amyloidosis.^3^ There are limited data on effectiveness for nodular amyloidosis.

## Conflicts of interest

None disclosed.
